# Impact of Metabolic Syndrome on the Prognosis of Ischemic Stroke Secondary to Symptomatic Intracranial Atherosclerosis in Chinese Patients

**DOI:** 10.1371/journal.pone.0051421

**Published:** 2012-12-10

**Authors:** Donghua Mi, Liqun Zhang, Chunxue Wang, Liping Liu, Yuehua Pu, Xingquan Zhao, Yilong Wang, Yongjun Wang

**Affiliations:** 1 Department of Neurology, Beijing Tiantan Hospital, Capital Medical University, Beijing, People’s Republic of China; 2 Neurology Department, St George’s Hospital, London, United Kingdom; University of Illinois at Chicago, United States of America

## Abstract

**Objectives:**

To analyze the effect of metabolic syndrome (MetS) on prognosis of ischemic stroke secondary to intracranial stenosis in Chinese patients.

**Methods:**

A prospective cohort of 701 patients with ischemic stroke, caused by intracranial stenosis, were followed at 3-month intervals for 1 year to monitor development of recurrent stroke or death. Imaging was performed using magnetic resonance angiography. MetS was defined using International Diabetes Federation (IDF) criteria.

**Results:**

MetS was identified in 26.0% of the cohort of stroke patients. Patients with MetS were more likely to be female, nonsmokers, and more likely to have a prior history of diabetes mellitus, high blood glucose and a family history of stroke than patients without MetS. During 1-year follow-up, patients with MetS had a non-significantly higher rate of stroke recurrence (7.1%) than patients without MetS (3.9%; P = 0.07). There was no difference in mortality (3.3% versus 3.5%, respectively). Multivariate Cox proportional hazards analysis (adjusting for gender, BMI, smoking, diabetes, and LDL-C) identified an association between that 1-year stroke recurrence and the presence of MetS (hazard ratio 2.30; 95% CI: 1.01–5.22) and large waist circumference (hazard ratio: 2.39; 95% CI: 1.05–5.42). However, multivariable analysis adjusting for the individual components of MetS found no significant associations between MetS and stroke recurrence. There were no associations between these parameters and mortality.

**Conclusions:**

Chinese patients with symptomatic intracranial atherosclerosis who have MetS, are at higher risk of recurrent stroke than those without MetS. However, MetS was not predictive of stroke recurrence beyond its individual components and one-year mortality.

## Introduction

Intracranial artery atherosclerotic stenosis is the principle cause of ischemic stroke in the Chinese population. The presence of symptomatic intracranial stenosis has been shown to account for 33% of cases of acute cerebral infarction and 51% of cases of transient ischemic attack (TIA) [1]. Annual stroke recurrence rates during the first year after the initial event were reported to be higher among patients with intracranial atherosclerosis (17.1%) than among those vascular lesions (10.9%) [2]. These data are supported by other studies showing high recurrent stroke rates among patients with intracranial arterial stenosis [3]–[5]. Recurrent stroke has been identified as a primary cause of death and disability for stroke survivors [6]. Recognition of patients at high risk of recurrence is, therefore, crucial for secondary stroke prevention.

Metabolic syndrome (MetS) comprises a constellation of abnormalities associated with increased risk for the development of type 2 diabetes and atherosclerotic vascular disease [7]. The prevalence of MetS among patients with symptomatic intracranial atherosclerotic disease is significantly higher than in the general population [8], [9]. Data from the Warfarin-Aspirin Symptomatic Intracranial Disease (WASID) trial suggest that MetS may not provide additional prognostic value beyond individual risk factors in patients with intracranial stenosis [9] However, a study from Taiwan suggests otherwise, and provides convincing data to indicate that MetS may indeed play an significant role in stroke recurrence [10].

Data on the prevalence of MetS in stroke patients with symptomatic intracranial stenosis mainland in China is lacking. The impact of MetS on the prognosis of these patients is also unknown. In this study we investigated whether the constellation of risk factors that define the MetS predict stroke recurrence more reliably than assessment of individual risk factors. The study was designed to evaluate the effect of MetS on stroke recurrence and mortality in a consecutive cohort of first-ever Chinese stroke patients with symptomatic intracranial stenosis.

## Methods

### Subjects

This was a hospital-based, prospective cohort study. Consecutive patients 18 years of age or older admitted to the tertiary hospital (between 1 June 2007 and 31 June 2009), within 7 days of onset of nondisabling ischemic stroke, were prospectively enrolled in the study. In all patients magnetic resonance angiography (MRA) showed the presence of 50–99% diameter stenosis in the internal carotid, middle cerebral, vertebral, or basilar artery. All patients presented with a. modified Rankin score ≤2. Patients with co-existing extracranial internal carotid stenosis (50–99%) in tandem with intracranial carotid or middle cerebral artery stenosis and those with non-atherosclerotic stenosis were excluded. None of the patients had a family history or previous history of atrial fibrillation as a potential source of cardiac embolism or antiphospholipid syndrome as a potential source of arterial clotting. Patients with contraindications to magnetic resonance imaging (MRI) including mechanical implanted pacemakers or other devices that would interfere with the MRI scan were also excluded.

The study protocol was approved by the institutional review board of Capital Medical University. Signed informed consent was obtained from the patients, spouses, immediate family members, caregivers or guardians.

### Cerebrovascular Evaluation

All patients were scanned within 1 week of symptom onset. The diagnosis of acute ischemic stroke was established by computer tomography (CT) and MRI. The extent of vascular stenosis was evaluated using MRA of the brain and/or carotid duplex. MRA data were three dimensional, time-of-flight images acquired from a Siemens Sonata (Erlangen, Germany) 1.5 T MRI scanner. The MRA images were read on a designated station by an experienced neuroradiologist who was blinded to the results of the clinical examination. The percentage of diameter stenosis of intracranial vessels was measured by visual inspection as previously described. Percent stenosis was calculated as [(1 − (D_stenosis_/D_normal_))] × 100, where D_stenosis_ was the diameter of the artery at the site of the most severe stenosis, and D_normal_ was the diameter of a normal proximal artery [11].

Duplex ultrasound of the extracranial carotid arteries was performed using Philips SD800 ultrasound machine with a 7.5-MHz transducer. The diagnostic criteria for the degree of carotid stenosis followed the definitions of the Society of Radiologists in Ultrasound [12].

### Outcome Assessment

All patients were followed up for 12 months, or until they reached the specified outcomes, i.e. had a second stroke or died. A standard telephone questionnaire was used to collect follow up data 3, 6, 9 and 12 months post-stroke. Caregivers provided answers on behalf of patients who were unable to speak.

The specified outcomes were the occurrence of a new episode of stroke (of any cause) or death. In cases where death occurred after a recurrent stroke, the event was classified as recurrence of stroke and not as death. New events requiring hospital admission were confirmed by reviewing the patient hospital records. Cases of suspected recurrent cerebrovascular event without hospitalization, were verified by a neurologist (YW). In all cases the cause of death was established from the death certificate. When no death certificate was available, the cause of death was classified as ‘unknown’.

Survival time was estimated from the onset of the initial stroke to the time of death. For those with recurrent of stroke, the survival time was the time from the initial stroke to the second stroke. For those lost to follow-up, the survival time was the interval from the initial stroke to the last follow-up.

### Definition of MetS

MetS was defined using previously published criteria from the International Diabetes Federation: [13]. The definition included the presence of central obesity (waist circumference ≥90 cm for Chinese men and ≥80 cm for Chinese women), plus any two of the following factors: triglycerides (TG) ≥150 mg/dL (1.7 mmol/L) or receiving specific treatment for elevated TG levels; high-density lipoprotein (HDL) cholesterol <40 mg/dL (1.03 mmol/L) in males and <50 mg/dL (1.29 mmol/L) in females, or receiving specific treatment for low HDL cholesterol; systolic blood pressure (BP) ≥130 mmHg or diastolic BP≥85 mm Hg, or treatment of previously diagnosed hypertension; fasting plasma glucose (FPG) ≥100 mg/dL (5.6 mmol/L) or previously diagnosed type 2 diabetes mellitus.

Waist circumference was measured between the lower rib margin and the iliac crest after a normal expiratory breath. TG, HDL-C, and fasting glucose levels were measured at the time of hospital admission. Plasma CRP levels were measured using the Ultrasensitive CRP kit (Cat. No. 68025) and control kit (Cat. No.68257) from Orion Diagnostics (Espoo, Finland). An automated assay procedure was conducted on a Hitachi 911 analyzer (NY, USA) according to the manufacturer’s instructions. Hypertension was based on sitting blood pressure measurements taken 14±3 days after the occurrence of stroke. Fasting glucose levels were obtained to avoid stress-related hyperglycemia, which often accompanies the acute phase of stroke. High body mass index (BMI) was defined as 25 kg/m^2^ [14].

### Statistical Analysis

All statistical analyses were performed using SAS software version 9.1.3. For univariate analyses, dichotomous or categorical variables were compared using the chi-square or Fisher’s exact test and continuous variables were compared using independent-sample t tests. Cox proportional hazards models were used to estimate the impact of multiple variables on the risk of death or stroke recurrence. Survival rates and stroke-free survival were estimated using the Kaplan-Meier product-limit method. All tests were two sided and values of P<0.05 were considered statistically significant.

## Results

### Baseline Characteristics

A total of 701 patients with first-ever symptomatic intracranial stenosis were enrolled in the study. The prevalence of MetS at admission was 26.0%. The baseline characteristics are summarized in Table 1. In the MetS group there were significantly more female patients and significantly more diabetic patients and patients with a family history of stroke than in the non-MetS group. However, there were fewer current smokers in the MetS group than in the non MetS group. Not surprisingly, BMI and high LDL-C levels were significantly higher in patients with MetS when compared to those of non-MetS. Fasting blood glucose was also higher in the patients with MetS (P = 0.0007). Stroke severity at admission based on the National Institutes of Health Stroke Scale (NIHSS), the degree of intracranial stenosis, C-reactive protein (CRP) and total cholesterol (TC) concentration were not significantly different between the two groups.

**Table 1:** Baseline characteristics and one-year outcomes.

	All (n = 701)	Non MetS (n = 519)	MetS (n = 182)	P-value
Gender (male), n (%)	454 (64.8)	360 (69.4)	94 (51.7)	<0.0001
Age (years), mean±SD	61.4±11.7	61.1±11.9	62.2±10.9	0.27
**Risk factors, n (%)**				
Hypertension	556 (79.3)	408 (78.6)	148 (81.3)	0.44
Diabetes mellitus	258 (36.8)	162 (31.2)	96 (52.8)	<0.0001
Hyperlipidemia	535 (76.3)	387 (74.6)	148 (81.3)	0.07
Family history of stroke	70 (10.0)	45 (8.7)	25 (13.7)	0.05
Current smoker	262 (37.4)	208 (40.1)	54 (29.7)	0.01
Heavy alcohol consumption*	38 (5.4)	32 (6.2)	6 (3.3)	0.14
Ischemic Heart disease	49 (7.0)	33 (6.4)	16 (8.8)	0.27
Peripheral vascular disease	3 (0.4)	1 (0.2)	2 (1.1)	0.17
**Laboratory data**				
High BMI	226 (32.2)	119 (22.9)	107 (58.8)	<0.0001
LDL-C, mmol/L	3.0±1.0	2.9±0.9	3.2±1.1	0.01
TC, mmol/L	4.8±1.3	4.7±1.1	4.9±1.5	0.06
CRP#	2.8 (1.0–9.2)	2.7(1.0–8.8)	3.1(1.2–9.3)	0.56
Fasting glucose, mmol/l	6.54±2.84	6.24±2.39	7.37±3.70	0.0002
NIHSS Score at admission#	5 (2–9)	5 (2–9)	5 (2–9)	0.96
**Degree of intracranial stenosis, n (%)**				0.6090
50–69%	192 (27.4)	137 (26.4)	55 (30.2)	
70–99%	137 (19.5)	103 (19.9)	34 (18.7)	
100%	372 (53.1)	(279 (53.8)	93 (51.1)	
**One-year outcomes**				
One-year stroke recurrence, n (%)	33 (4.7)	20 (3.9)	13 (7.1)	0.07
One-year death, n (%)	24 (3.4)	18 (3.5)	6 (3.3)	0.91

BMI body mass index; CRP C-reactive protein; LDL-C low-density lipoprotein cholesterol; MetS metabolic syndrome; NIHSS NIH Stroke Scale; TC total cholesterol.

* Heavy alcohol consumption was defined as more than 4 units per day.

# Variables are median (quartile range).

### MetS and Risk of Stroke Recurrence

**Figure 1:**
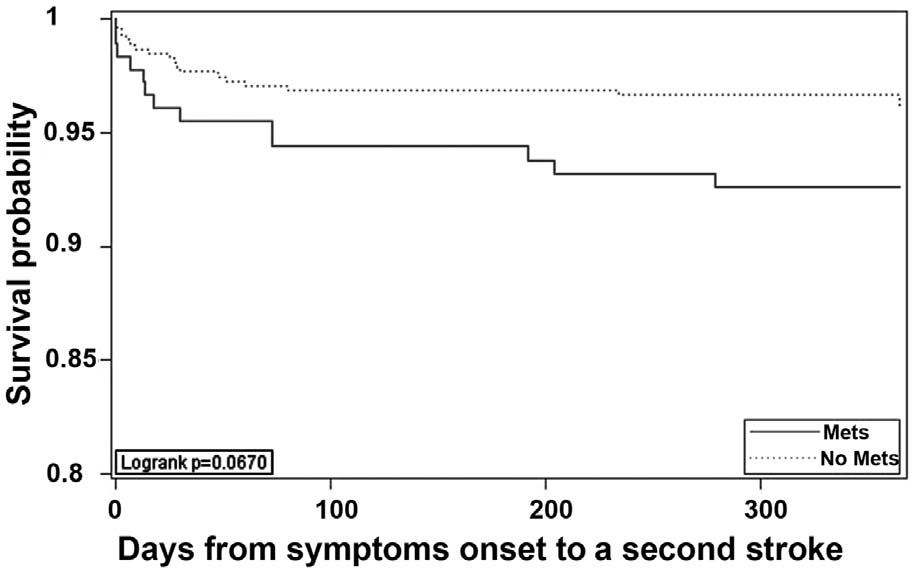
Cumulative probability of survival free from stroke.

Patients with MetS had a higher rate of recurrent stroke (7.1%) than those without MetS (3.9%) but the difference was not statistically significant when tested using univariate methods (P = 0.07; Table 1). The time to stroke recurrence was also shorter in patients with MetS than patients without MetS (Figure 1) but again the difference was not statistically significant (P = 0.07).

Multivariate Cox proportional hazards analysis, adjusting for gender, high BMI, current smoking, history of diabetes and LDL-C (Table 2) identified MetS as being associated with 1-year stroke recurrence (HR: 2.30; 95% CI: 1.01–5.22). Of the five components of the MetS, central obesity (elevated waist circumference) was associated with an increased risk of stroke recurrence (HR: 2.39; 95% CI: 1.05–5.42) after adjustment for the same factors. Table 3 shows the results of multivariate analysis adjusting for the individual components of MetS. When this procedure was followed no association was found between stroke recurrence and the presence of MetS or high waist circumference (Table 3).

**Table 2:** Hazard Ratios (95% CIs) showing the relationship between MetS and its individual components with stroke recurrence and death^a^.

	N (%)	Stroke recurrence		Death	
		Multivariate Adjusted HR	P-value	Multivariate AdjustedHR	P-value
MetS	182 (26.0)	2.30 (1.01–5.22)	0.0465	0.96 (0.33–2.82)	0.9416
**International Diabetes Federation criteria ** [**12**]					
Elevated waist circumference	213 (30.4)	2.39 (1.05–5.42)	0.0372	1.50 (0.55–4.05)	0.4252
Elevated TG	274 (39.1)	1.25 (0.58–2.70)	0.5652	0.88 (0.34–2.26)	0.7906
Low HDL-C	374 (53.4)	1.25 (0.58–2.67)	0.5698	1.025 (0.43–2.46)	0.9563
Elevated BP^b^	655 (93.4)	-	0.9853	0.66 (0.15–2.87)	0.5768
Elevated fasting blood glucose*	373 (53.2)	1.56 (0.66–3.67)	0.3102	0.97 (0.38–2.47)	0.9456

Multivariable models were adjusted for sex, smoking behavior, diabetes, high BMI and LDL-C.

BP blood pressure; TG triglyceride; HDL-C high-density lipoprotein cholesterol; HR hazard ratios.

a Multivariate Cox regression analysis of stroke and death outcome in patients with ischemic stroke caused by symptomatic intracranial atherosclerosis.

b There were too few non hypertensive patients in the study to enable the impact of this variable to be analyzed.

* adjusted for sex, smoking behavior, high BMI and LDL-C.

**Table 3:** Hazard ratios of stroke recurrence and death for MetS and individual components in the model adjusted for metabolic syndrome and its individual components^a^.

	Stroke recurrence		Death	
	Multivariate Adjusted HR	P-value	Multivariate Adjusted HR	P-value
Metabolic syndrome	0.75 (0.13–4.21)	0.7416	0.52 (0.08–3.29)	0.4869
**International Diabetes Federation criteria ** [**12**]				
Elevated waist circumference	3.36 (0.61–18.66)	0.1654	2.53 (0.45–14.16)	0.2924
Elevated TG	1.32 (0.59–2.91)	0.4994	1.24 (0.50–3.11)	0.6404
Low HDL-C	1.10 (0.50–2.42)	0.8164	1.08 (0.45–2.63)	0.8587
Elevated BP^b^	–	0.9901	0.72 (0.16–3.1)	0.6591
Elevated fasting blood glucose	1.59 (0.64–3.93)	0.3193	1.02 (0.38–2.70)	0.9731

a Multivariate Cox regression analysis of stroke and death outcome in patients with ischemic stroke caused by symptomatic intracranial atherosclerosis.

b There were too few non hypertensive patients in the study to enable the impact of this variable to be analyzed.

Multivariable models were adjusted for sex, smoking behavior, diabetes, high BMI, LDL-C and individual components of MetS.

BP blood pressure; TG triglyceride; HDL-C high-density lipoprotein cholesterol; HR hazard ratios.

### MetS and Risk of Mortality

**Figure 2:**
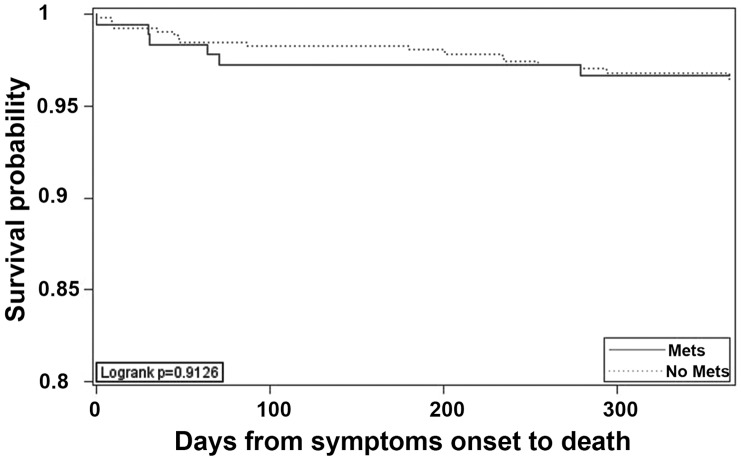
Cumulative probability of survival free from death of any cause.

As shown in Table 1, the 1-year mortality rate in patients with MetS (3.3%) was similar to that in patients without MetS (3.5%; P = 0.91). The time to death was not significantly different between the two groups (Figure 2, P = 0.91). Multivariate Cox proportional hazards analyses, adjusting for gender, smoking behavior, diabetes, high BMI and LDL-C found no association between the presence of MetS and 1-year mortality.

## Discussion

Racial and ethnic differences influence individual propensity for cerebrovascular atherosclerosis. Intracranial cerebrovascular stenosis have been shown to be more common in Asians, Hispanics and African Americans [15]–[18] than in other people groups. The morbidity associated with intracranial atherosclerotic stenosis in Chinese patients with stroke and TIA is has been estimated to be between 40% and 50% [19].

The prevalence of MetS has continued to increase worldwide. Studies reported between 2002 and 2008 indicate that approximately 24% of adult Americans and 25% of adult Europeans have MetS [20], [21]. Although only 11% of adult Chinese were reported to have MetS in 2002 [22], the prevalence of MetS in China is steadily rising. However, it is currently unknown what percentage of Chinese stroke patients with symptomatic intracranial stenosis also have MetS. This is the first prospective cohort study to evaluate the prevalence of MetS among Chinese patients with first-occurrence symptomatic stroke caused by intracranial stenosis and to investigate its effect prognosis in terms of stroke recurrence and mortality.

We showed that 182 of the 701 patients (26.0%) with symptomatic intracranial stenosis who entered the study had MetS. This is lower than the prevalence of MetS reported in Korean patients with intracranial stenosis (55.2%)and lower than in an American population reported in the WASID study (43%) [7], [8]. The difference in prevalence may be due to differences in definitions used to define MetS. The Korean and WASID studies applied the criteria from the National Cholesterol Education Program-Adult Treatment Panel III (NCEP-ATP) [23], which was intended to identify people at long-term high risk for atherosclerotic vascular disease and included insulin resistance as a hierarchically equal criterion. However, the IDF definition used in our study considered insulin resistance to be a common physiological mechanism that causes the clustering of metabolic abnormalities that contribute to MetS [24].

Consistent with previous findings [25], we found that there were more female patients in MetS group, and a higher rate of diabetes, indicating a possible association between diabetes and MetS. We also observed that patients with MetS were more likely to have a family history of stroke than those without the MetS. The mechanism underlying this finding is uncertain.

The incidence of recurrent stroke within the first 12 months among patients with MetS (7.1%) was almost double that in patients without MetS (3.9%). We showed that the presence of MetS or a large waist circumference was associated with 1-year stroke recurrence independent of gender, high BMI, current smoking, diabetes and low density lipoprotein. However after adjusting for the individual components of MetS the association was no longer present. In the WASID study [9] differences in time to ischemic stroke during a mean follow-up of 1.8 years (HR 1.7; 95%; CI: 1.1–2.6; P = 0.012) were also dependent on individual factors within the definition of MetS. however, the post-hoc analysis of the WASID data may have been limited by the small sample size, and non-fasting estimation of baseline glucose and triglyceride levels.

An interesting finding from our study was that central obesity, the primary condition for MetS in the IDF criteria, was associated with stroke recurrence. To date, the pathophysiology of MetS seems to be largely attributable to insulin resistance and it is thought that central obesity may have a pivotal role in the development of insulin resistance [26].

A growing body of evidence predominantly from population-based studies or from studies in cardiovascular patients, suggests that MetS is associated with a significant increased risk of all-cause mortality [27]. Wong and Li showed that patients with intracranial atherosclerosis, especially with coexisting extracranial carotid disease, were at increased risk of death or recurrent vascular events [2]. They also showed that vascular disease burden, advanced age, diabetes, atrial fibrillation, and previous stroke were predictive of a poor outcome. Our study provides further evidence to suggest that MetS may be associated with early recurrent stroke in stroke patients secondary to intracranial stenosis. However, no evidence in our study has shown that MetS is associated with increased risk of mortality.

The main limitation of our study is the use of MRA as diagnostic tool to confirm the diagnosis of intracranial stenosis, instead of gold standard, conventional angiography. This may have exaggerated the degree of stenosis. In addition, the number of patients with normal blood pressure was very small (46 patients) which meant that we were not able to investigate the relationship between hypertension and stroke recurrence. The relative short follow-up interval may also limit the scope of our results.

### Conclusion

To our knowledge, this is the first large scale prospective study on the effect of MetS on stroke patients with intracranial stenosis in Mainland China. The study was based at major stroke center in this region and provided important data on early stroke recurrence rate and mortality among patients with symptomatic intracranial stenosis concurrent with MetS. We showed that patients with MetS may be at increased risk for early stroke recurrence than those without MetS. We also identified, central obesity as a potentially important predictor of stroke recurrence in patients with intracranial stenosis. However, MetS was not predictive of stroke recurrence beyond its individual components and one-year mortality after the first-ever stroke caused by intracranial stenosis.
